# Changes in attitudes and behaviors supportive of maternal and newborn health in Ethiopia: an evaluative case study

**DOI:** 10.1186/s12884-021-03865-8

**Published:** 2021-05-28

**Authors:** William T. Story, Yared Amare, Lara M.E. Vaz, Heather Gardner, Halkeno Tura, Gail Snetro, Mary V. Kinney, Steve Wall, Abeba Bekele

**Affiliations:** 1 Department of Community and Behavioral Health, University of Iowa, Iowa City, IA 52242 USA; 2 Independent Consultant, Addis Ababa, Ethiopia; 3 Save the Children US, Washington, DC, 20001 USA; 4 Ethiopia Country Office, Save the Children International, Addis Ababa, Ethiopia

**Keywords:** Ethiopia, Maternal, newborn, and child health, Community-based, Demand creation, Evaluation, Case studies

## Abstract

**Background:**

Ethiopia’s high neonatal mortality rate led to the government’s 2013 introduction of Community-Based Newborn Care (CBNC) to bring critical prevention and treatment interventions closer to communities in need. However, complex behaviors that are deeply embedded in social and cultural norms continue to prevent women and newborns from getting the care they need. A demand creation strategy was designed to create an enabling environment to support appropriate maternal, newborn, and child health (MNCH) behaviors and CBNC. We explored the extent to which attitudes and behaviors during the prenatal and perinatal periods varied by the implementation strength of the *Demand Creation Strategy for MNCH-CBNC*.

**Methods:**

Using an embedded, multiple case study design, we purposively selected four kebeles (villages) from two districts with different levels of implementation strength of demand creation activities. We collected information from a total of 150 key stakeholders across kebeles using multiple qualitative methods including in-depth interviews, focus group discussions, and illness narratives; sessions were transcribed into English and coded using NVivo 10.0. We developed case reports for each kebele and a final cross-case report to compare results from high and low implementation strength kebeles.

**Results:**

We found that five MNCH attitudes and behaviors varied by implementation strength. In high implementation strength kebeles women felt more comfortable disclosing their pregnancy early, women sought antenatal care (ANC) in the first trimester, families did not have fatalistic ideas about newborn survival, mothers sought care for sick newborns in a timely manner, and newborns received care at the health facility in less than an hour. We also found changes across all kebeles that did not vary by implementation strength, including male engagement during pregnancy and a preference for giving birth at a health facility.

**Conclusions:**

Findings suggest that a demand creation approach—combining participatory approaches with community empowering strategies—can promote shifts in behaviors and attitudes to support the health of mothers and newborns, including use of MNCH services. Future studies need to consider the most efficient level of intervention intensity to make the greatest impact on MNCH attitudes and behaviors.

**Supplementary Information:**

The online version contains supplementary material available at 10.1186/s12884-021-03865-8.

## Background

Ethiopia is one of five countries accounting for the greatest number of maternal and neonatal deaths globally. Maternal mortality rates (298 deaths per 100,000 live births) and neonatal mortality rates (28 deaths per 1000 live births) remain among the highest in the world [1, 2]. Many of these deaths are preventable through access to high quality care before, during, and after pregnancy. Specifically, interventions around the time of birth could avert 54% of maternal deaths, 71% of newborn deaths, and 33% of stillbirths in low- and middle-income countries [3]. Despite efforts to increase use of and access to maternal and newborn health care in Ethiopia, contact with health services remains low [4]. Only 74% of women reported at least one antenatal care (ANC) contact with a skilled provider, 43% reported four or more ANC contacts, 50% reported that their last birth was with a skilled birth attendant, and 34% reported their newborn received a postnatal check within the first 2 days of birth [4]. Poor use of maternal and neonatal health services is attributed to economic, social, and cultural barriers [5].

### Attitudes and behaviors associated with the risk of mortality

Studies attribute the causes of maternal and newborn deaths in Ethiopia to factors ranging from biomedical (e.g., infections, hemorrhage, eclampsia, birth complications) and economic (e.g., distance to health facility, lack of transportation, cost), to social and cultural norms [6]. Social and cultural norms—perceived standards of acceptable attitudes and behaviors within formal and informal networks [7]—are connected to risk of maternal and newborn mortality in a multitude of ways, including hindering maternal and newborn health service utilization [5, 8]. Women may form opinions and engage in specific behaviors that could negatively affect their health and the health of their newborn because of perceived consequences of not conforming to social norms and how others in their social network are behaving.

In Ethiopia, attitudes and behaviors have been shown to be associated with low maternal and newborn service utilization. Some of these factors include negative attitudes around male engagement during pregnancy, late disclosure of pregnancy status, and the belief that small or sick newborns are too weak to survive [9–17]. Such factors lead to late initiation of ANC, deliveries at home by untrained providers, and delayed care seeking for newborn complications [18–22], which can have consequences for maternal and newborn morbidity and mortality.

To address attitudes and behaviors that are often deeply embedded in social and cultural norms, it is critical to gain a better understanding of how these norms place women and newborns at greater risk during pregnancy, childbirth, and immediately following childbirth. Multilevel strategies that integrate actions and provide structural and systems support at national, zonal, district, and community levels could provide the potential for change. Specifically, engaging communities in ways that challenge individual’s expectations regarding normative behaviors can have a positive effect on the deep-rooted norms adversely affecting maternal and newborn health [8].

### The Demand Creation Strategy for Maternal, Newborn, and Child Health - Community-Based Newborn Care (MCNH-CBNC) 

In 2014, Save the Children conducted formative assessments and found a diverse array of social and cultural barriers preventing women and newborns from getting necessary care, such as seclusion of newborns for 1 to 2 months after birth, the belief that newborn illnesses have supernatural causes that require traditional cures, and the perception that care for newborns is unavailable at health facilities [23]. The Federal Ministry of Health (FMOH) and partners developed a demand creation strategy aiming to reduce newborn and child mortality by addressing social and cultural norms through Primary Health Care Units (PHCUs), the Health Extension Program, and Kebele Command Posts (KCPs)—an existing, multi-sectoral community platform used for decision-making [24]. Strategy approaches included improving MNCH- and CBNC-related household practices and norms, increasing timely care-seeking for maternal and newborn illnesses, and creating enabling social norms to support appropriate MNCH and CBNC behaviors.

The *Demand Creation Strategy for MNCH-CBNC* (hereinafter referred to as the *Demand Creation Strategy*) addresses changes at the individual, household, community, health system, and national policy levels. A combination of community empowering and systems strengthening approaches at each of these levels were posited to have an effect on individual-level MNCH household practices and care-seeking behaviors, and, ultimately, reductions in morbidity and mortality (for more details on the *Demand Creation Strategy*, see [4]).

From 2015 to 2017, Save the Children worked in 21 zones across four regions to integrate the *Demand Creation Strategy*. KCPs were strengthened by diversifying and expanding membership to include individuals from the Health Development Army (HDA), traditional birth attendants in a non-delivery role, faith-based leaders, school leaders and other community leaders, and people affected by maternal or newborn death. In addition to expanding membership, Save the Children strengthened the capacity of the KCPs to organize around MNCH-CBNC, develop community action plans, and act collectively. Save the Children also developed and used an on-the-job training package to strengthen capacity of FMOH zonal, woreda (i.e., district), PHCU, and community partners (including faith-based organizations) to implement community-empowering demand creation approaches [25]. These demand creation approaches were prioritized by communities to address identified barriers and encourage solutions to reduce maternal and neonatal mortality, including male engagement, early disclosure of pregnancy and use of ANC, institutional deliveries, belief that a small/sick newborn can survive, and care-seeking for sick newborns.

### Evaluation objectives

The objective of this evaluation was to explore the extent to which the *Demand Creation Strategy* contributed to attitude and behavior changes across the prenatal (i.e., pregnancy) and perinatal (i.e., childbirth and immediately following childbirth) periods. Specifically, we examined whether the following changes during the prenatal and perinatal periods varied by the implementation strength of the *Demand Creation Strategy*: (1) male engagement during pregnancy, (2) early disclosure of pregnancy status, (3) early use of ANC, (4) institutional deliveries, (5) belief that small or sick newborns can survive, and (6) care seeking for newborn complications. We focused our evaluation design and data collection procedures to respond to these six areas of inquiry.

## Methods

### Evaluation design

We used an embedded, multiple case study design to evaluate the impact of the *Demand Creation Strategy* by collecting qualitative data at different implementation levels (e.g., individual, household, and community). We employed a case study design to be able to generalize the lessons learned from the case studies, also known as analytic generalization. Analytic generalization is based on advancing theoretical concepts that have been built into the design of the case studies, which will allow us to make theoretical contributions to similar programs [26]. An embedded design allowed us to assess the *Demand Creation Strategy* across multiple cases by collecting various forms of data for each case, including key informant interviews, groups interviews, focus group discussions (FGDs), in-depth interviews, and illness narratives. Each case was a kebele, which is the smallest administrative unit in Ethiopia, similar to a village or neighborhood. We chose the kebele to represent each case because the majority of the demand creation interventions were delivered at the kebele-level through the KCP. The multiple case study approach (rather than a single case study) allowed us to compare cases (or kebeles) by implementation strength of the *Demand Creation Strategy*. Our sampling strategy for the case study is described below.

### Site selection and sampling

Ethiopia is divided into the following administrative units (from largest to smallest): regions, zones, woredas (or districts), and kebeles. The evaluation focused on three zones embedded in two regions—one zone in Oromia Region and two zones in Southern Nations Nationalities and Peoples Region (SNNPR)—because these zones represented the areas in which the *Demand Creation Strategy* had been implemented for the longest period of time. Based on the resources available to conduct an in-depth analysis of the *Demand Creation Strategy* at multiple levels, we limited our evaluation to four kebeles using a three-step purposive selection process, a common sampling procedure used in qualitative research [27]. First, we selected one zone from each region with Save the Children staff available from the *Demand Creation Strategy* project. Second, within each zone, we purposively selected one woreda that met the following criteria: (1) geographic accessibility, (2) availability of staff who are familiar with the *Demand Creation Strategy*, (3) uninterrupted program implementation throughout the life of the project, (4) adequate variability among KCPs in terms of implementation strength, and (5) socio-cultural representativeness of the entire project. Third, within each woreda, we purposively selected two kebeles with varying levels of demand creation implementation through the KCP—one KCP with low and one with high implementation strength. The term “implementation strength” has been used to describe the supply- and demand-side conditions required to ensure effective coverage of an intervention [28], which we defined as the intensity [29] or extent to which inputs and processes are in places and interventions actually implemented [30]. To determine the level of implementation strength, we applied a set of 14 criteria ranging from engagement of religious leaders to application of participatory tools for demand creation (see Supplemental Table 1). A score of 6 or lower was considered high implementation strength, and a score greater than 6 was considered low implementation strength.

Although the unit of analysis for each case was the kebele, the population within each case included men and women from multiple levels, including zone, woreda, kebele (village), and household. Multiple levels of data collection allowed us to understand the context in which the *Demand Creation Strategy* was implemented and how it affected individual norms and practices within each kebele. We first identified respondent groups based on their contribution to the outcomes of the *Demand Creation Strategy* and their potential to contribute to the objectives of this evaluation, then purposively selected individuals within each group to be interviewed. Overall, 150 individuals agreed to participate in the evaluation (Table 1).

**Table 1:** Number of study participants by type and by Kebele

Data collection method and participant type	Oromia Region Zone A, Woreda 1		SNNP Region Zone B, Woreda 2	
	High IS Kebele	Low IS Kebele	High IS Kebele	Low IS Kebele
Key informant interview, regional Save the Children Demand Creation coordinator	1		1	
Group (or key informant) interview, staff of Woreda Health Office	1		4	
Group interview, members of the PRT of health center serving kebele	4	3	3	3
Key informant interviews, kebele HEWs	1	1	2	1
FGD, members of Strengthened KCP	6	12	8	7
FGD, kebele HDA members	7	4	6	7
FGD, fathers of newborns	5	6	4	4
FGD, mothers-in-law of mothers of newborns	4	5	3	3
In-depth interviews, mothers of infants born in prior 6 months	5	5	5	5
Illness narratives with mothers (and their families) of infants who had an illness in neonatal period and survived	Not Available	Not Available	7 (4 narratives)	7 (4 narratives)

*FGD* Focus Group Discussion, *HDA* Health Development Army, *IS* Implementation Strength, *PRT* Performance Review Team

### Data collection

Data collection took place from June to July in 2017 as part of a formal evaluation of the *Demand Creation Strategy*. Two male and two female interviewers participated in a six-day training, which included a field pre-test of the qualitative interview guides. The team spent from 8 to 10 days collecting qualitative data in each of the four kebeles. A detailed list of the data collection method, respondent type, and number of respondents from each group is in Table 1. Interview guides were developed in English and translated into Amharic and Afan Oromo. Each respondent was asked to consent to the interview and audio recording before the start of each interview. The informed consent process included an explanation of the purpose, methods, and potential risks and benefits of participation; a statement about the voluntary nature of the interview; and a statement about the confidentiality of all information shared during the interview. Following consent, the team audio recorded all interviews and FGDs; each interview was simultaneously translated and transcribed verbatim into English after the completion of the interview.

### Analytic strategy

The two principal investigators (PIs)—one in Ethiopia and one in the US—developed a codebook for data analysis based on the interview guides. The PIs used the codebook to each independently code transcripts from one low and one high implementation strength kebele each using NVivo 10.0. Once the coding was complete, the PIs wrote individual case reports. The findings presented below represent a cross-case analysis of the four reports, which describes the similarities and differences between the low and high implementation strength kebeles according the six areas of inquiry described in our evaluation objectives.

## Results

The findings from this evaluation are presented in order of the six areas of inquiry, starting with three during the prenatal period—male engagement during pregnancy, early disclosure of pregnancy status, and early use of ANC—and ending with three during the perinatal period—institutional deliveries, belief that small or sick newborns can survive, and care seeking for newborn illnesses. Each section starts with the differences between high and low implementation strength kebeles and ends with the common characteristics among all kebeles*.*

### Prenatal period

#### Male engagement during pregnancy

There were no noticeable differences in male engagement and support by implementation strength. Across all kebeles, most mothers and husbands reported that husbands provided emotional and instrumental support to their wives during and after their pregnancy. One of the most common forms of support mentioned was help with household chores, such as fetching wood and water, and cooking, including arranging for hired help to do the same.

“I encourage her when she feels weak, I tell her that there is not as much risk of bleeding when she delivers at the health center as there is at home. I also support her by working on different things that she has been working on at home, like fetching water. I prevent her from lifting heavy items because the fetus’ position might be affected and it is not good for her health.”

*28-year-old husband, high implementation strength kebele*

Husbands also encouraged their wives to eat well and some cooked or bought food for their wives. Some men accompanied or encouraged their wives to attend ANC and to deliver at a health facility, but others did not. There was also broad support and expectations for male involvement in decisions and actions related to MNCH at the community level across all kebeles.

“I: What is the trend in your community, do men really participate in decision making in [MNCH] issues?

R1: Yes, we discuss and decide, and we give her money if she has to go alone, and if she needs someone to go with her, she will be accompanied.

R2: To add on this, in our area if someone refuses to go with his sick wife and child to the health center, he is less respected. Because [we live in] a competition era when men do what they have seen in the neighborhood. If someone gives good care to his wife and children, the others will do the same.”

*21-year-old husband (R1) and 45-year-old husband (R2), low implementation strength kebele*

#### Early disclosure of pregnancy status

Respondents in low implementation strength kebeles were more likely to state disadvantages to early disclosure of pregnancy compared to high implementation strength kebeles. In high implementation strength kebeles, there have been changes in the last 2 years regarding early disclosure of pregnancy status, especially among mothers and their husbands. However, these changes were not mentioned in low implementation strength kebeles.

“It differs from family to family [regarding disclosure to husband]. In previous times, it was when the pregnancy was visible that a husband may know she is pregnant, but today there is no problem. Both husband and wife know when her menstruation stops.”

*32-year-old HDA, high implementation strength kebele*

Across all kebeles, most husbands, HDA members, and mothers-in-law agreed that it was important to discuss pregnancy when menstruation stops or when she feels the fetus moving. The timing of this varied across groups from as early as the first month to as late as the sixth month of pregnancy. Advantages of discussing pregnancy early included support from the husband and others close to the mother, such as helping with chores, reducing her workload, ensuring that she is eating well, attending ANC, and emotional support (especially from the husband). Disadvantages of disclosing pregnancy early included embarrassment for the mother as well as fear that she could lose the child after telling others that she was pregnant.

#### Early use of ANC

Husbands, mothers-in-law, and HDA members mentioned that ANC should start early, with a slight difference between high and low implementation strength kebeles. In high implementation strength kebeles, most agreed that ANC should start around the third month; in low implementation strength kebeles, most agreed that the fourth or fifth month is ideal. Support for early ANC has also increased in recent years due to the work of health extension workers (HEWs), HDA leaders, and other groups. These changes were mentioned more frequently in the high implementation strength kebeles compared to the low implementation strength kebeles.

“It has completely changed. She gets support from the family. They tell her that her life is important to them. They say it is when you survive that we can survive. The family encourages her to go for ANC. The community itself has changed; they support each other.”

*27-year-old HDA, high implementation strength kebele*

Across all kebeles, husbands, mothers-in-law, and HDA members mentioned a number of benefits of starting ANC early, including the ability to check on the health of the mother and baby, to check the position of the fetus, to prevent abortion, and to promote the health and growth of the baby. Mothers mentioned these advantages as well as the ability to get medications and vaccines and to get advice on nutrition and facility delivery.

### Perinatal period

#### Institutional deliveries

There was no difference in delivery location preference between high and low implementation strength kebeles. In all kebeles, mothers, fathers, and mothers-in-law believed that a health facility was the best place to give birth. Mothers-in-law across all kebeles mentioned that they would advise mothers to deliver at a health facility. Most of the respondents specifically mentioned a health center (woreda level) as the preferred place of delivery due to proximity to their homes. Some of the respondents mentioned the hospital (regional level) as their preference due to its capacity to handle complicated pregnancies. Reasons for preferring a health facility delivery over a home delivery included: (1) preventing maternal and newborn death; (2) the ability to treat complications, such as bleeding, a retained placenta, high blood pressure, and asphyxia; (3) better equipment, such as incubators for newborns and intravenous fluids for the mother; and (4) better care, including safe and clean delivery practices, delaying bathing of the newborn, and providing folic acid and vaccines.

“The traditional delivery may harm the neonate and the mother. She may lose blood. There may be a retained placenta and she may die until we reach the health center. So, to prevent all these dangers, it is better to deliver at a health center.”

*24-year-old husband, low implementation strength kebele*

In one kebele, the HDA members mentioned that there is now a 500–1000 Birr ($15–31 USD) punishment for delivering at home, which was established by the local *idir*—an informal financial association. Other changes in knowledge and attitudes related to institutional delivery were mostly attributed to the government providing education through the HEW.

#### Belief that small or sick newborns can survive

In low implementation strength kebeles, mothers-in-law were more fatalistic and believed that a newborn’s survival depended on the will of God compared to high implementation strength kebeles. Across all kebeles, most mothers and husbands believed a newborn would survive if they get sick or are very small at birth. They also agreed that sick newborns should be taken to a health center, hospital, or doctor who can treat them.

“Yes, [they will be willing to seek treatment]. It was in previous times that they said, ‘He is too small. He cannot handle the medication.’ But, nowadays, everybody knows and they will take them to where there is treatment.”

*23-year-old mother, high implementation strength kebele*

In some kebeles (irrespective of implementation strength), barriers to newborn care included a lack of knowledge of the services available at health facilities as well as the cultural and religious ceremony of having the newborn blessed by a local spiritual leader (known by some as *hamachisa*^1^) only after which s/he can go to the doctor. However, some respondents added that they no longer use traditional treatments or practice traditional customs in the same way when seeking care for newborn illnesses. One husband described how the practice of *hamachisa* is changing. For some families with a sick newborn, *hamachisa* is now a symbolic gesture before continuing on to the health center.

“*Hamachisa* is still there. Newborns are taken there just to give it as ‘savior’ for the baby when sick, and for other ceremonies. But still, the families can give the money (2-5 birr) and take the baby to the health center and bring the baby afterwards. There are some changes.”

*28-year-old husband, high implementation strength kebele*

#### Care seeking for newborn illnesses

There was a difference in knowledge about care-seeking practices between the high and low implementation strength kebele, based on illness narratives with mothers and family members of infants who became sick and survived. Three out of four mothers in the low implementation strength kebele reported that they did not receive any information about newborn illness, whereas all four mothers in the high implementation strength kebele reported receiving education about newborn illness from health workers or HEWs.

“They told me [about illness signs] at the hospital and when the HEW came home to see my baby a week after the delivery she told me, ‘If your baby has a rash, a lesion on the umbilicus or breathing problems, call me. I have the medication for her and if it is beyond me, you will go to health center.’”

*20-year-old mother, high implementation strength kebele*

In the high implementation strength kebele, only one mother took longer than a day to seek care once she noticed the symptoms, whereas three mothers took longer than a day to seek care in the low implementation strength kebele. While the amount of time it took families to travel to the facilities was similar (approximately 12 h), there were differences across kebeles in the time it took to receive care once the mother arrived at the health facility. In the high implementation strength kebele, all four mothers waited less than an hour to receive care, whereas mothers in the low implementation strength kebele waited between 1 and 4 h.

Across all kebeles, husbands and mothers-in-law agree that sick newborns should be taken to the health facility. Several fathers and mothers-in-law asserted that care seeking for sick newborns was uncommon a few years ago and that people were more likely to take sick newborns to local healers or to undertake prayer ceremonies instead. The respondents also mentioned that health education provided by the HEW and other community groups brought about these changes.

“[Taking newborns to the health facility if s/he is sick] is a recent trend. For example, if the baby has influenza, they [used to] say it is just minor and stay at home, and they don’t go to the health center. But now the community has learned to take sick babies to the health center.”

*28-year-old husband, high implementation strength kebele*

## Discussion

This evaluation provides a unique contribution by examining the effect of a package of demand creation strategies on household and community attitudes as well as care seeking and household behaviors. Further, we were able to observe the differences and similarities in attitudes and behaviors by the level of implementation strength, which indicates the potential influence of the *Demand Creation Strategy* beyond the underlying temporal trends.

### Prenatal period

#### Male engagement during pregnancy

Male engagement during the prenatal period can help expedite decisions to seek care and reduce the time it takes to get to the hospital, which are causes of increased maternal and neonatal mortality [32]. Prior studies in Ethiopia have shown that supportive male involvement is associated with health care utilization, namely ANC attendance and institutional delivery [10–12, 15]. Our results show that male partners provided substantial support to their female partners during pregnancy, including the provision of nutritious food, assisting with domestic work, and supporting their use of ANC. Our findings also demonstrate that the community not only supports male involvement in decision-making regarding MNCH-CBNC, but also expects them to get involved, which indicates a shift in social norms. The findings regarding male engagement did not differ by implementation strength and were in contrast with the prior evidence from Ethiopia that reported low male engagement in MNCH [33].

#### Early disclosure of pregnancy status

Early disclosure of pregnancy can help ensure a safe and healthy pregnancy [34], which can lead to lower risk for childbirth complications and adverse birth outcomes. However, cultural beliefs in some parts of Ethiopia prevent women from disclosing their pregnancy in the first trimester, making it difficult to initiate ANC early [13, 16]. Our findings varied by implementation strength; respondents in the low implementation strength kebeles mentioned barriers to early disclosure of pregnancy, whereas respondents in high implementation strength kebeles mentioned positive changes regarding early disclosure of pregnancy. Across all implementation areas, there was agreement about the importance of early disclosure of pregnancy citing advantages, such as support from husbands and close family members. Although it is common for women to hide pregnancies or wait to tell others about their pregnancy in sub-Saharan Africa [13, 16], the topic of early disclosure of pregnancy is understudied.

#### Early use of ANC

The timing of ANC is an essential component of maternal health services, which helps with early detection, management, and prevention of problems during pregnancy as well as other services necessary for a healthy pregnancy [35]. On average, only 20% of mothers in Ethiopia start their initial ANC visit during the first trimester [36] due to a multitude of barriers, such as the perception that pregnancy is not a serious issue, lack of support from parents and spouses, and stigma associated with early initiation of ANC [19, 22]. Although the timing of ANC was slightly earlier in high compared to low implementation strength kebeles, there was substantial agreement among respondents that ANC should start early. Our results show that the *Demand Creation Strategy*—especially the work of HEWs, HDA leaders, and other community groups—influenced the timing of ANC.

### Perinatal period

#### Institutional deliveries

Prior data suggests that only 28% of women in Ethiopia gave birth with a skilled provider [36]. Most women in rural Ethiopia choose to stay home during childbirth because there are inadequate resources at health facilities as well as common barriers, such as distance, transportation, cost, and even disrespect and abuse [37, 38]. Our results demonstrate that respondents (including mothers, fathers, and mothers-in-law) across all kebeles prefer to give birth at a health facility, which aligns with temporal changes at the national level [4]. Most preferred health centers due to their proximity to the community, whereas others preferred hospitals because they have the capacity to handle complicated pregnancies. Belay and Sendo [39] found similar results in northwest Ethiopia, where women reported a preference for health facilities due to the ability to manage complications during labor or delivery. We also found that there is a disincentive to staying home during childbirth, as some respondents reported that there is a fine of up to 1000 Birr for delivery at home. This finding is consistent with other research in Ethiopia [18]. Preferences for institutional deliveries appear to be common across all kebeles; however, it is not clear if this is due to an external policy or normative change due to the project.

#### Belief that small or sick newborns can survive

In Ethiopia, beliefs about the danger of newborn illness vary from views about pathogens to spirituality [40]. Our findings regarding the belief that small or sick newborns can survive varied by implementation strength. For example, mothers-in-law in low implementation strength kebeles were more fatalistic and believed that a newborn’s survival depended on the will of God. However, mothers and husbands across all kebeles believed that small or sick newborns can survive and should be taken to a health facility. The fatalistic view of newborn survival is consistent with prior studies in Ethiopia, where it has been reported that little is done to help low birth weight babies survive and the cause of perinatal death is often attributed to supernatural forces [9, 14, 41]. However, our findings suggest that these attitudes are shifting, especially among mothers and fathers. In addition, we found that the traditional ceremony of *hamachisa* appears to be changing in areas where it was once practiced. Although there is little documentation of this religious practice in the literature, our findings suggest that local traditions can be changed by challenging social norms that are harmful to newborns.

#### Care seeking for newborn illnesses

In Ethiopia, there are specific care seeking practices for different types of newborn illness, which are driven by social norms and local conceptualizations of illness. The use of home-based herbal remedies (e.g., feeding newborns butter and herbal drinks) or consultation with traditional healers as a first point of treatment, can lead to delays in appropriate neonatal care [42, 43]. In addition, other cultural beliefs that lead to delays in care-seeking have been reported, such as the seclusion of newborns to protect from evil eyes and harmful spirits [43]. Our findings show that knowledge about care seeking for newborn illness, delays in care seeking, and delays in receiving care varied by implementation strength. Mothers in low implementation strength kebeles reported receiving less information about newborn illness and experiencing longer delays in seeking and receiving care for their sick newborn compared to those in the high implementation strength kebeles. Across all kebeles, many of the respondents said that previously families would take sick newborns to local healers or to undertake prayer ceremonies, instead of health professionals or para-professionals (e.g., HEWs). This change in care seeking behaviors for sick newborns (especially in the high implementation strength areas) suggests perceptions of newborn illness can be changed in a relatively short period of time when communities are engaged and take ownership.

### Limitations of the evaluation

This evaluation was not without limitations. We were only able to select four cases for the evaluation. Rather than increase the number of cases, we opted to examine the evaluation objectives in greater depth by interviewing a variety of stakeholders and informants within each case. Although small sample size does not allow us to generalize our results to other populations in Ethiopia, our evaluation design allows for analytic generalization, permitting us to advance theoretical concepts built into the design of the case studies [26]. Further, our study design only provides evidence for the contribution of the *Demand Creation Strategy* (especially in high implementation strength kebeles), but not necessarily attribution of program effects.

Woreda health officials and the Save the Children Program Coordinators independently ranked kebeles’ implementation strength using the 14 criteria; rankings were validated through a consensus process with project staff. The distinction between high and low implementation strength, however, was not always clear as scores sometimes varied by small differences; therefore, kebeles selected for inclusion in the study were from the score extremes to ensure differences between high and low implementation strength.

## Conclusion

This evaluation demonstrates that attitudes and behaviors related to MNCH-CBNC can change in a short amount of time with different levels of demand creation implementation strength. During the prenatal period, male engagement activities were successful independent of implementation strength, whereas success with changing attitudes around early disclosure of pregnancy and early ANC was related to greater implementation strength. During the perinatal period, we found that changes in preference for institutional delivery were independent of implementation strength, whereas perceptions of newborn survival and positive care-seeking behaviors were related to greater implementation strength. Future studies and program evaluations need to consider the most efficient level of intervention intensity to make the greatest impact on MNCH norms and behaviors.

## Supplementary Information

**Additional file 1.**

## Data Availability

The datasets generated and/or analysed during the current study are not publicly available due to the qualitative nature of the data, but are available from the corresponding author on reasonable request.

## Abbreviations

ANC: Antenatal care
CBNC: Community-based newborn care
EPHI: Ethiopian Public Health Institute
FGD: Focus group discussion
FMOH: Federal Ministry of Health
HDA: Health Development Army
HEW: Health Extension Worker
KCP: Kebele Command Post
MNCH: Maternal, newborn, and child health
PHCU: Primary Health Care Unit
PI: Principal investigator
PRT: Performance Review Team
SNNPR: Southern Nations Nationalities and Peoples Region
USD: United States Dollar
WHO: World Health Organization

## Footnotes

1 In some parts of Ethiopia, *hamachisa* is a traditional religious system of belief governed by traditional spiritual leaders (also known as *Aba Ayantu*) [31]. The belief system centers on the *hamachisa* ceremony at which the *Aba Ayantu* blesses the infant and names him/her. This usually takes place around 40 days after the birth for a boy and 80 days for a girl. Until the baby has been blessed, the mother and baby live in seclusion and no one except the mother may touch the baby.
