# Supplementary material for: Changes in attitudes and behaviors supportive of maternal and newborn health in Ethiopia: an evaluative case study
Source: BMC Pregnancy Childbirth. 2021 May 28;21:407. doi: 10.1186/s12884-021-03865-8 (PMC8161997; doi:10.1186/s12884-021-03865-8)
Supplement: Supplementary file 1 — Additional file 1. [file 12884_2021_3865_MOESM1_ESM.docx]

| **Supplementary Table 1: Implementation Strength Criteria** | |
| --- | --- |
| **Instructions**: Put "1" instead of “X” if each statement is true for the Kebele Command Post (KCP). Higher scores equal lower implementation strength. | |
| **Criteria Number** | **Criteria statement** |
| 1 | Has no religious leaders engaged |
| 2 | Has no TBAs engaged |
| 3 | Has no affected populations engaged |
| 4 | KCP re-structured/reformed by government |
| 5 | Meetings without quorum |
| 6 | Does not have updated meeting minutes recorded in the last two months |
| 7 | No actions identified in meeting |
| 8 | Not meeting regularly as per their schedule (excluding special seasons such as harvesting time) |
| 9 | Do not receive regular support from the health center on demand creation implementation |
| 10 | No meetings/activities held in community |
| 11 | Pregnant Women’s Conferences not conducted regularly as per the schedule |
| 12 | Community action plan not complete |
| 13 | Has not applied participatory tools |
| 14 | Has not raised human, financial or material resources |
